# Inclusion body myositis in a patient with rheumatoid arthritis

**DOI:** 10.1093/rap/rky046

**Published:** 2018-11-05

**Authors:** Mohammad Tariq, Anurag Bharadwaj

**Affiliations:** Rheumatology Department, Basildon and Thurrock University Hospital, Basildon, UK

**Key message**

- Inclusion body myositis is rarely associated with RA and should be looked for.

Sir, The inflammatory myopathies are rare conditions and include PM, DM, necrotizing autoimmune myositis and IBM. IBM is insidious, follows a slowly progressive course and involves predominantly the distal muscles [1]. It is most common after the age of 50 years [2], with a male to female ratio of ∼3:1 [3].

The diagnosis of IBM is based on the clinical, neurophysiological and histopathological findings, but MRI study of skeletal muscle may contribute to the diagnosis. Myositis-specific antibodies are much less frequent in IBM compared with DM and PM [4].

The detection of an active myositis is uncommon in autoimmune diseases, and IMB has been reported, in single patients, in the presence of SLE and SS [5], SSc, autoimmune cholangitis and chronic thyroiditis [6], and four IBM patients with RA have been reported [5, 7].

Treatment of sporadic IBM remains a challenge. IBM is generally refractory to treatment with CSs, immunosuppressants or IVIg, although long-term randomized controlled trials are lacking. Cases of IBM in patients with RA have rarely been reported. IVIg treatments are considered as therapeutic options for IBM patients, and some controlled studies have shown a clinical response in up to 25% of cases with high-dose IVIg administration [8]. Current evidence suggests that immunosuppressive drugs, including CSs, are ineffective in IBM, although long-term randomized controlled trials are lacking.

We present a 53-year-old male with long-standing seropositive RA who did not respond adequately to synthetic standard disease-modifying anti-rheumatic drugs. This included MTX and SSZ, and the failure two anti-TNF agents and rituximab. He also had a history of pulmonary embolism and hypertension. His medications included MTX, prednisolone, atorvastatin, warfarin and candesartan. He was reviewed in the rheumatology clinic, with a history of tiredness and weakness in his shoulders and hands. There was no history of dysphagia or breathing difficulty. There was no family history of primary muscle disorder. Power in the flexors and extensors of the wrist and fingers was 4/5, quadricep femorus +3/5, and ankle dorsiflexion was 4/5. There was no evidence of skin rash. Neurological examination, including reflexes and sensations, was normal.

His alanine aminotransferase was 82 U/l (normal <50 U/l) and creatine kinase was 396 U/l (normal <320 U/l). His ANA and ANCA were negative, but RF and anti-CCP antibodies were strongly positive. Thyroid function test and electrolytes were normal. CRP was 5 mg/l (normal <5 mg/l). HBV and HBC screen and HIV test were negative. His aCL antibodies were high, with IgG 87 GPL U/ml (normal is 0.5–9.9 gplu/ml) and B2 glycoprotein-1 IgG 121 u/ml (normal is 0.0–10.0 u/ml). US scan revealed no biliary tract pathology. EMG was requested, which confirmed features of mild myopathy in the biceps muscles. MRI of the thigh confirmed muscle oedema and inflammation. PET CT revealed no evidence of malignancy.

A muscle biopsy showed characteristic findings, including severe cytoplasmic inflammation in the endomysium, basophilic granular inclusions, rimmed vacuoles and occasional eosinophilic inclusions. Myositis screen was negative.

Our patient was diagnosed with myositis after receiving anti-TNF agents, but discontinuing these agents had no effect on muscle strength. In the literature there is a case report of inflammatory myositis as a result of infliximab therapy. A few cases of IBM associated with various autoimmune disorders have been described. It is not clear whether IBM in our patient was induced by anti-TNF therapy or related to RA. He was already on rituximab therapy, but it did not help with his arthritis and myositis.

Although IBM does not respond to CSs, based on the clinical situation a decision was taken to treat with oral prednisolone 40 mg/day, which was tapered gradually to 10 mg/day with no further deterioration in muscle strength. He was commenced on tocilizumab, which controlled his arthritis but showed no effect on his muscle strength. He was referred for physiotherapy and rehabilitation. His last creatine kinase was 317 U/l.

*Funding*: No specific funding was received from any bodies in the public, commercial or not-for-profit sectors to carry out the work described in this manuscript.

*Disclosure statement*: The authors have declared no conflicts of interest.
